# End Stage Renal Disease—A Nephrologist’s Perspective of Two Different Circumstances as Typified by Kidney Transplantation Experience in a Nigerian Hospital Versus a Large US Medical School

**DOI:** 10.3390/healthcare5030031

**Published:** 2017-07-11

**Authors:** Macaulay Amechi Chukwukadibia Onuigbo

**Affiliations:** 1 Department of Medicine, Mayo Clinic College of Medicine, Rochester, MN 55905, USA; onuigbo.macaulay@mayo.edu; Tel.: +1-715-838-3891; Fax: +1-715-838-1946; 2 Department of Nephrology, Mayo Clinic Health System, Eau Claire, WI 54702, USA; 3 College of Business, University of Wisconsin MBA Consortium, Eau Claire, WI 54701, USA

**Keywords:** end stage kidney disease, Garki Hospital, Abuja, Nigeria, hemodialysis, peritoneal dialysis, public-private partnership (PPP), quality of life, renal replacement therapy (RRT), United States of America (USA), University of Maryland Medical School (UMMS)

## Abstract

Renal transplantation is the sine qua non consummate form of renal replacement therapy (RRT) for end stage renal disease (ESRD). Despite the increasing ESRD burden worldwide, developing countries continue to experience a gross lack of RRT options for its teeming citizens with ESRD. This report is a demonstration of a nephrologist’s experience and dilemma trying to make sense of the yawning disparity between RRT options, especially renal transplantation, as it applies to the citizens of the USA versus the citizens of Nigeria. The limited three-year experience of renal transplantation at Garki Hospital, located in Abuja, the capital of Nigeria, which is one of the very few centers carrying out renal transplantation in Nigeria, was starkly contrasted with this author’s first-hand experience at the University of Maryland Medical School, in Baltimore, Maryland, USA, as a Nephrology Fellow between 2000 and 2002. The potential role of public-private partnership (PPP) ventures in developing countries is considered as a way to help bridge this gap.

Prologue:

Just the other day, in late April 2017, during weekly scheduled hemodialysis rounds at one of the Mayo Clinic Dialysis Services outpatient hemodialysis units in Northwestern Wisconsin, this author was rounding on a pleasant 76-year old Caucasian widow who had been on dialysis since June 2016 for end stage renal disease (ESRD). Her other past medical history was notable for statin-induced myopathy, dyslipidemia, hyperuricemia, and gout, as well as peripheral vascular disease with previous bilateral femoral artery bypass procedures, a left knee meniscus repair, and unilateral renal artery stenosis, which was stented in November of 2013 and re-stented again in December of 2014. During the hemodialysis rounds, she had been asked how she was doing. “Today is my last day,” she had chuckled with a broad smile. She was getting a living kidney transplant in two days’ time at Mayo Clinic, Rochester. “The donor is my 49-year old daughter!” she added with an even broader smile. “I hope all goes well,” this author had intoned. “It will. It will,” she added, with glee.

This tells the whole story; for the ESRD patient on thrice weekly hemodialysis, or any other renal replacement therapy (RRT) option for that matter, the act of getting a kidney transplant is a transformative phenomenon. “It is as if being born again,” one of my patients had noted previously.

## 1. Introduction—Kidney Transplantation Is the Sine Qua Non Consummate form of Renal Replacement Therapy for End Stage Renal Disease

In an investigation by Eggers, nearly 30 years ago, the costs of maintaining patients with functioning renal allografts were found to be only one third that of maintaining patients on dialysis and, because the quality of life is usually much better, renal transplantation is causing a convergence of the best clinical and economic outcomes for patients with end stage renal disease [1,2]. The relative risk of death during the first two weeks after transplantation was 2.8 times as high as that for patients on dialysis who had equal lengths of follow-up since placement on the waiting list, but at 18 months the risk was much lower (relative risk, 0.32; 95% confidence interval, 0.30 to 0.35; *p* < 0.001) [2]. The likelihood of survival became equal in the two groups within five to 673 days after transplantation in all the subgroups of patients we examined [2]. The long-term mortality rate was 48 to 82% lower among transplant recipients (annual death rate, 3.8 per 100 patient-years) than patients on the waiting list, with relatively larger benefits among patients who were 20 to 39 years old, white patients, and younger patients with diabetes [2].

In a 1986 analysis by Krakauer, despite the initial costs of renal transplantation, three years after renal transplantation the cost of maintaining kidney transplants are paid back when compared to matched patients on maintenance hemodialysis [1,3]. Three years after renal transplantation, the patients with renal allografts represented a net savings to the US Medicare program [1,3].

From the abovementioned investigations, it is no wonder that kidney transplantation is globally adjudged to represent the best form of (renal replacement therapy) RRT for end stage renal disease [1,2,3,4,5,6]. Nearly 75% of the transplant recipients were able to work, compared with between 24.7 and 59.3% of the patients undergoing dialysis. Furthermore, on three subjective measures (life satisfaction, well-being, and psychological affect) transplant recipients had a higher quality of life than patients on dialysis [4]. Transplantation was considerably less expensive during the second year after transplantation [5]. Over the two years, transplantation was both more effective and less costly than dialysis [5]. This was true for all subgroups of patients examined, including patients older than 60 and diabetics. The conclusion was that renal transplantation was more effective and less costly than dialysis in all subgroups of patients examined [5].

## 2. Worldwide Prevalence and Incidence of End Stage Renal Disease (ESRD) Versus Access to RRT

A recent Lancet systematic review of worldwide access to treatment for end stage renal disease demonstrated that, in 2010, 2.618 million people received RRT worldwide, whereas the estimated number of patients needing RRT was between 4.902 million (95% CI 4.438–5.431 million) in the conservative model and 9.701 million (8.544–11.021 million) in the high-estimate model [7]. The implication of these derivations is that at least 2.284 million people might have died prematurely because RRT could not be accessed [7]. Only in four countries—Japan, Singapore, Taiwan, and the USA—is RRT known to be provided to almost all individuals needing it; for at least another sixteen countries, about half of people needing RRT receive it [7,8,9]. Nevertheless, in Africa, less than 20% of people needing RRT receive it [7,10,11]. The majority of patients with end stage renal disease (ESRD) perish because of lack of funds, as very few can afford regular maintenance dialysis and renal transplantation is often not available [10]. Dialysis rationing resulting from limited facilities and healthcare personnel in low- and middle-income countries such as South Africa must be addressed on several fronts [11]. In Ghana, for example, each session costs more than $100, which is far out of reach for most people [11]. Very clearly, there is a direct and correlational relationship between RRT prevalence and individual national per capita income [7,12]. 

## 3. Renal Transplantation Experience in Nigeria—A Case Analysis

With a specific emphasis on access to renal transplantation as an RRT option, the picture is even more so the worse for it vis a vis the yawning disparity between developed rich countries and undeveloped/developing countries [7,12,13,14,15,16]. 

The first reported renal transplantation in Nigeria was carried out at the College of Health Sciences, Obafemi Awolowo University, Ile-Ife, Nigeria, on June 26, 2002. A living related kidney donation between two brothers occurred, in addition to the immunosuppression regimen with Cyclosporine, Prednisolone, and Azathioprine [13]. In Nigeria, between 2000 and 2010, a total of 143 renal transplantations were performed in five transplant centers, some of which had only recently opened [14]. One-year graft and patient survival was 83.2% and 90.2%, respectively, while the five-year graft and patient survival was 58.7% and 73.4%, respectively. Mortality was reported in 38 (27%) of recipients [14]. Renal transplantation in Nigeria led to an improved survival but was bedeviled by unaffordability, inaccessibility, a shortage of donor organs, and poor legislative support [14]. This was a call for the enactment of relevant organ transplant legislation, subsidization of renal care, and further development of local capacities to improve renal transplantation utilization and thus lead to better outcomes in Nigeria [14]. Earlier, Bamgboye had posited that renal transplantation rates in the developing world (as with other modalities of renal replacement therapy) are considerably lower than in the developed world [15]. Identified reasons for the paucity of renal transplantation offerings in Nigeria included poverty, low education levels of the populations of these countries, the absence of functional dialysis and transplant units with adequately trained and motivated staff, and the lack of appropriate health policies derived from renal registry data [15]. Suggested measures to improve the quality of care should center around improvement of the socioeconomic and political scenario in these countries [15]. As of December 2014, there were six active public and two private kidney transplant programs in Nigeria that had been in place for one to 14 years [16].

In a Lancet Commentary, Coresh and Jafar acknowledged that although government budgets are unlikely to accommodate universal access to RRT in most low-income and middle-income countries, innovative public-private partnerships (PPP) can work [12]. Excellent examples of such successful PPP ventures providing affordable and accessible RRT options abound in Southeast Asia, including hemodialysis, peritoneal dialysis, and renal transplantation, with partners ranging from ordinary citizens to rich multimillionaire philanthropists, charitable not-for-profit foundations, and business organizations [17].

In Nigeria, the first PPP venture to start renal transplantation initiated in Garki Hospital, Abuja, located in the capital city of the country, Abuja [18]. The three-year experience of renal transplantation at this health institution was recently presented by this author at the recent World Congress of Nephrology, the biennial conference of the International Society of Nephrology held in Mexico City, Mexico in April 2017 [18].

**Figure 1:**
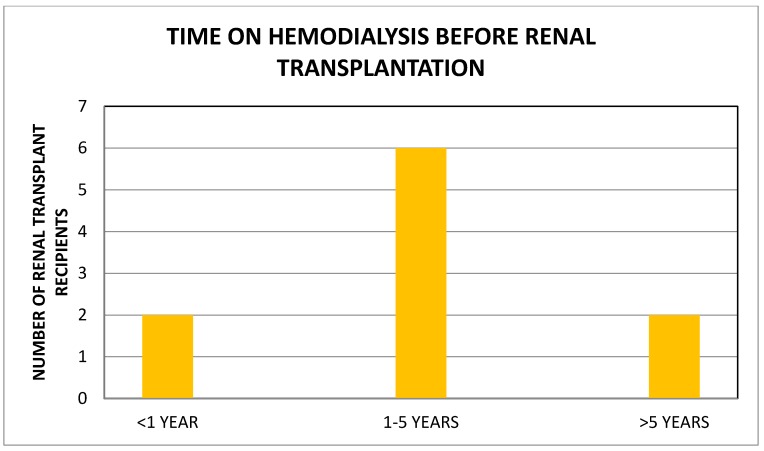
Time on hemodialysis before renal transplantation.

**Figure 2:**
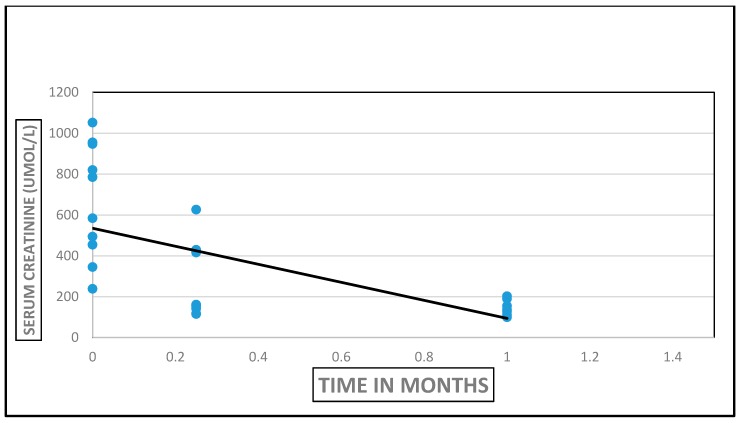
Serum creatinine trajectory from transplantation to one-month post-transplantation in all 10 renal transplant recipients.

Garki Hospital, Abuja, Nigeria is a rejuvenated PPP medical facility in the nation’s capital that started renal transplantation in late 2013. We completed a retrospective analysis of all available medical records of patients who underwent renal transplantation at the center between 2013 and 2016 [18]. There were 10 renal transplant recipients, 5 males and 5 females, with a median age of 48 (19–64) years, all on maintenance hemodialysis for a median period of 39 (1–192) months before renal transplantation (Figure 1). All renal allografts were living kidney allografts—2/10 were living-related and 8/10 were living-unrelated donors including two wives. Hypertension (8/10) was the most common cause of ESRD. Cross-match testing was performed at Hammersmith Hospital, London. Donor nephrectomies were by the finger-assisted technique. There was “bundling” of renal transplantation procedures—two renal transplantation procedures were performed on same day during three different periods to maximize the utilization of transplant manpower and resources. Induction immunosuppression used Thymoglobulin/Methyl Prednisone and maintenance immunosuppression utilized Tacrolimus/Mycophenolate Mofetil/Prednisone. Hospital LOS was 23 (12–34) days. Immediate renal transplant complications were acute pulmonary edema (2), peri-allograft hematoma (2), and wound dehiscence (1). The results of renal transplantation were excellent by serum creatinine assays (Figure 2). As at the end of 2016, acute rejection was absent in 7/10, probable in one and data was not available in another two renal transplant recipients [18]. Four patients died, and clinical information on the cause of death is lacking. The latest mean serum creatinine in the six surviving renal transplant recipients is 1.38 mg/dL at a median of 5 (1–13) months post-transplantation [18].

The conclusion from this retrospective analysis of the renal transplantation in this PPP venture was that the facilitation of renal transplantation in third world countries remains a major public health dilemma, as thousands of ESRD patients do not have affordable access to renal transplantation [18]. We demonstrated that sustainable renal transplantation can become more available to Nigerians with the application of more of such PPP opportunities [18]. This is a call on the Nigerian governments, both at the federal and state levels, all well-meaning wealthy Nigerian philanthropists, non-Nigerian multimillionaire and multibillionaire philanthropists, as well as NGOs around the world to assist well-established health facilities such as Garki Hospital, Abuja, Nigeria, to do even more to help the terrible and harrowing plight of the very needy and underserved Nigerian ESRD patients [18].

## 4. Conclusions and Prospectives

By 1998, the number of kidney transplants performed at the University of Maryland Medical School (UMMS) had increased yearly from 51 in 1991 to 285 in 1998 [19]. Then, at UMMS over the past three years, the increase in the number of kidney transplants was ascribed almost exclusively to a marked increase in living donor transplants, from 49 cases in 1995 to 130 cases in 1998; a 160% increase [19]. By the time this author was completing his Nephrology Fellowship at UMMS between the years 2000 and 2002, the total number of kidney transplant procedures carried out at that center had ballooned to about 1000 a year [20,21,22]. That meant that UMMS in those years was the busiest renal transplant center, worldwide—this translated to nearly three renal transplants a day. Indeed, this author easily remembers long days at the hospital as a Nephrology Fellow on the Transplant Service, when as many as three kidney transplantation procedures were successfully completed in a 24-hour cycle.

These renal transplantation statistics from UMMS stand out in stark contrast with this author’s experience visiting Garki Hospital, Abuja, Nigeria, in April 2016, to work with the renal transplant team at this institution to review possible areas of collaboration and cooperation in order to facilitate more renal transplantation procedures there [18]. With increased governmental and non-governmental support and involvement in PPP ventures, together with all the suggested methodologies discussed above to improve the availability, affordability, and accessibility of RRT options in Nigeria including renal transplantation, we believe that the best is yet to come in Nigeria [14,15,16,18].

Clearly, there is the need for more functional and sustainable renal transplant centers in Nigeria. Strategies to enable these objectives include the establishment of PPP programs as Garki Hospital, Abuja, the facilitation by the various governments in Nigeria to enable the extensive professional expertise among Nigeria’s health professionals in Nigeria to help contribute assiduously to this effort, and the establishment by non-profit NGOs of top-class laboratories and other ancillary support systems, as well as the upgrade of existing Federal University Teaching Hospitals that have generally fallen behind in their renal transplantation programs as a result of poor funding from the federal Government.

The Garki Hospital experience was the result of a private consortium bidding for and the achievement of a time-limited lease of the facilities at the former federal government General Hospital, and it’s transformation into a very respectable and functional medical institution that still serves the general public but with more enhanced medical specialty service such as renal transplantation. This center could indeed do more with better funding, and we call upon well-meaning Nigerian and foreign philanthropists to invest in such centers to expand the coverage of renal transplantation services in Nigeria. 

The high mortality rate of up to 40% in this three-year experience is most concerning. The inability of Nigerians to consistently have affordable and easy access to medications including transplant immunosuppressive therapy, the absence of a national health insurance policy, the relative higher prevalence of infections, concerns about the availability of prompt therapeutic drug monitoring, and other diagnostic services including pathology diagnosis, and indeed the inconsistency of prompt access to medical attention when needed are all contributory factors to this unacceptable high mortality among renal transplant recipients. It is this author’s wishes and prayers that hopefully as Nigeria’s healthcare system improves, through which many of these problems would begin to be resolved.
